# Extraspinal Type I Dural Arteriovenous Fistula with a Lumbosacral Lipomyelomeningocele: A Case Report and Review of the Literature

**DOI:** 10.1155/2015/526321

**Published:** 2015-04-08

**Authors:** Khaled M. Krisht, Michael Karsy, Wilson Z. Ray, Andrew T. Dailey

**Affiliations:** Department of Neurosurgery, Clinical Neurosciences Center, University of Utah, Salt Lake City, UT 84132, USA

## Abstract

Seven cases of adult spinal vascular malformations presenting in conjunction with spinal dysraphism have been reported in the literature. Two of these involved male patients with a combined dural arteriovenous fistula (DAVF) and lipomyelomeningocele. The authors present the third case of a patient with an extraspinal DAVF and associated lipomyelomeningocele in a lumbosacral location. A 58-year-old woman with rapid decline in bilateral motor function 10 years after a prior L4-5 laminectomy and cord detethering for diagnosed tethered cord underwent magnetic resonance imaging showing evidence of persistent cord tethering and a lipomyelomeningocele. Diagnostic spinal angiogram showed a DAVF with arterial feeders from bilateral sacral and the right internal iliac arteries. The patient underwent Onyx embolization of both feeding right and left lateral sacral arteries. At 6-month follow-up, MRI revealed decreased flow voids and new collateralized supply to the DAVF. The patient underwent successful lipomyelomeningocele exploration, resection, AV fistula ligation, and cord detethering. This report discusses management of this patient as well as the importance of endovascular embolization followed by microsurgery for the treatment of cases with combined vascular and dysraphic anomalies.

## 1. Introduction 

Spinal dural arteriovenous fistulas (DAVFs) constitute 80% of all spinal arteriovenous malformations while dysraphic abnormalities of the neuraxis are the most common congenital malformations of the central nervous system [1, 2]; however, their combined occurrence is exceedingly rare [3]. Seven adult cases of spinal lipomas/lipomyelomeningoceles with an associated vascular malformation (AVM or DAVF) have been reported [4–10], with only 2 involving an extraspinal DAVF with an associated lipomyelomeningocele [7]. This is the third reported case of a joint lipomyelomeningocele and DAVF.

## 2. Case Presentation

A 58-year-old woman was referred for neurosurgical evaluation because of a 2-year history of rapid decline in bilateral motor functioning. Ten years earlier, she underwent an L4 and L5 laminectomy for cord detethering at an outside facility but had no meaningful improvement. Since then, she had experienced a gradual deterioration in her ambulation. Magnetic resonance imaging (MRI) demonstrated persistent evidence of cord tethering, with an L4/L5 lipomyelomeningocele extending to the sacral hiatus. Notably, prominent flow voids were also seen within the ventral subarachnoid space, with the most caudal draining vessel extending below the S2 sacral body. She had 3/5 strength in her left leg, 4/5 strength in her right, globally diminished sensation to light touch, and pin prick, as well as some bladder urgency. The patient did endorse some lower back pain but also has a history of chronic lower back pain. A diagnostic spinal angiogram showed evidence of a DAVF with arterial feeders from the bilateral lateral sacral arteries and right internal iliac artery with venous drainage to a dilated perimedullary vein. The patient underwent Onyx embolization of both feeding right and left lateral sacral arteries.

Three months later, the patient exhibited marked improvement in her leg strength and sensation, improved 4-5/5 strength throughout her lower extremities, improved walking with assistance, and marked improvement in balance. At her 6-month follow-up, however, she reported stagnant functional capacity, with worsening of her left leg function that began after a ground-level fall. Physical examination revealed stable to improved right leg strength with subtle worsening of left leg strength (3-4/5). MRI revealed decreased flow voids compared with those seen on the prior study with no evidence of a new abnormality; however, one month later, evaluation revealed globally decreased lower extremity strength (3/5). Repeat spinal angiogram and lumbar MRI revealed new collateralized supply from the right internal iliac artery and lateral sacral branches. The DAVF now extended into the lipomyelomeningocele with dilated draining veins rostrally and spinal canal involvement.

**Figure 1:**
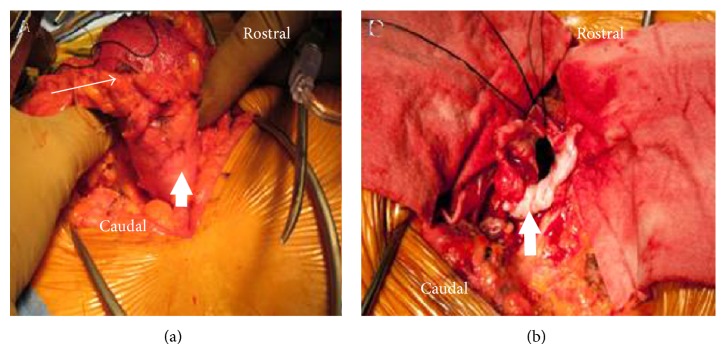
(a) Intraoperative photograph showing the grossly abnormal fatty myelomeningocele (long arrow) adherent to the dura (small arrow). (b) Intraoperative photograph after the circumferential dissection of the lipomyelomeningocele from the surrounding dura (small arrow).

The findings of redeveloping DAVF and the patient's clinical condition prompted us to recommend lipomyelomeningocele exploration/resection, DAVF ligation, and cord detethering. A standard midline approach was utilized, and a grossly abnormal fatty myelomeningocele was circumferentially dissected (Figure 1(a)). Intradural exploration demonstrated diffuse blood supply tracking through the lipomyelomeningocele with a large draining vein ventral to a tethered filum. The filum was sectioned, and the DAVF was clipped and ligated well into the arterialized vein. The remaining lipomyelomeningocele was mobilized and resected (Figure 1(b)).

**Figure 2:**
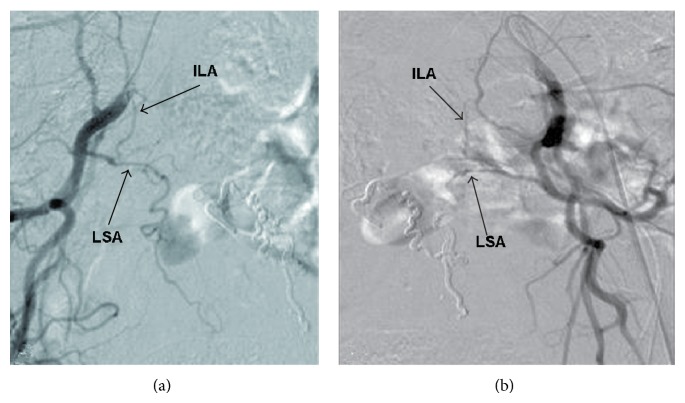
Spinal angiogram through right (a) and left (b) internal iliac artery injections demonstrating complete obliteration of the dural arteriovenous fistula with no evidence of draining vein. ILA: iliolumbar artery; LSA: lateral sacral artery.

Six months after surgery, the patient regained full motor function of her proximal lower extremities with 5/5 strength throughout and 5/5 and 4/5 strength in her distal right and left lower extremities, respectively. MRI of the thoracic spine demonstrated marked improvement in the appearance of the spinal DAVF, with the tortuous vessels no longer evident. Follow-up spinal angiogram showed no evidence of residual DAVF or early draining vein (Figure 2).

## 3. Discussion

An extensive literature search yielded only 4 reported cases of adults presenting with a combined congenital AVM and myeloschisis anomaly along with 2 pediatric cases [4, 8–11]. Therefore, the existence of both anomalies simultaneously is a very rare congenital event. This rare cooccurrence may be precipitated by the focal maturation of the pluripotent mesoderm, which migrates between the two disjoined ectodermal layers and predominantly matures as a lipoma within the mesodermal primitive vascular plexus that subsequently fails to form its capillary component [8, 9]. Ehni and Love [12] proposed that the mesenchymal cells forming the primordial vascular plexus may also give rise to adipocytes. Differentiation of adipocytes is suppressed by neural crest cells under normal conditions of development; however, if the neural crest cells are defective, inhibition fails and a lipoma may arise with an AVM forming secondarily.

Even rarer than the coexistence of an AVM with a myeloschisis is a congenital lipomeningocele with an acquired DAVF. Given the late, gradual onset of symptoms with slowly developing neurological deficits manifesting in adulthood, it has been suggested that spinal DAVFs are acquired lesions. All reported cases of combined spinal DAVF and dysraphism were discovered at a relatively advanced age (40–53 years) with symptoms attributable to a developing vascular anomaly. There have only been three other described adult cases of combined DAVF and myeloschisis [6, 7]. Our case is the third reported case of lipomyelomeningocele and DAVF and the first reported case in a female patient (Table 1). There is a general consensus that spinal DAVFs are slow-growing acquired vascular lesions secondary to thrombosis with a late clinical presentation that predominantly occur in men [13, 14]. Type I DAVFs, as in our case, have a high flow/low pressure shunt with a rare chance for rupture. The delayed neurological worsening is believed to be due to venous congestion and/or steal phenomena, which result in cord ischemia in both instances [6]. They usually are found at the level of the lower thoracic cord or conus [7]. Their slow-growing nature and their rare association with other congenital anomalies argue against a congenital basis for DAVF formation. It is unclear whether the lipomeningocele, which precedes the DAVF formation based on its congenital origin, lends to increased predisposition for extraspinal venous thrombosis and subsequent formation of DAVF or whether those are two distinct entities that may coincidentally occur together. Our case confirms that formation of DAVF with spinal dysraphisms can occur in both men and women and can contribute to significant patient morbidity but can be managed well with surgical and endovascular treatment.

## Figures and Tables

**Table 1:** Adult cases of combined dural arteriovenous fistula and dorsal myeloschisis.

Author (year)	Sex/age (years)	Combined lesions
Rice and Jelsma (1986) [9]	F/23	AVM and lipoma
Djindjian et al. (1989) [6]	M/53	DAVF and lipoma
Chatkupt et al. (1993) [4]	F/20	AVM and myelomeningocele
König et al. (1999) [7]	M/50	DAVF and lipomyelomeningocele
Lee et al. (2000) [8]	M/44	AVM and lipomyelomeningocele
Weon et al. (2005) [10]	M/30	AVM and lipomyelomeningocele
Erdogan et al. (2007) [5]	M/40	DAVF and lipomyelomeningocele
This paper	F/58	DAVF and lipomyelomeningocele

AVM: arteriovenous malformation; DAVF: dural arteriovenous fistula.
